# Chemical Investigation and Regulation of Adipogenic Differentiation of Cultivated *Moringa oleifera*

**DOI:** 10.3390/ph17101310

**Published:** 2024-10-01

**Authors:** Duc Dat Le, Eunbin Kim, Thinhulinh Dang, Jiseok Lee, Choon Ho Shin, Jin Woo Park, Seul-gi Lee, Jong Bae Seo, Mina Lee

**Affiliations:** 1 College of Pharmacy and Research Institute of Life and Pharmaceutical Sciences, Sunchon National University, 255 Jungangno, Suncheon 57922, Jeonnam, Republic of Korea; ddle@scnu.ac.kr (D.D.L.); dangnhulinh02051998@gmail.com (T.D.); 2 Department of Biomedicine, Health & Life Convergence Sciences, BK21 Four, Biomedical and Healthcare Research Institute, Mokpo National University, Muan 58554, Jeonnam, Republic of Korea; dmsqls0749@naver.com (E.K.); amuretart@naver.com (J.L.); jwpark@mokpo.ac.kr (J.W.P.); 3 Suncheonman Moringa Union, Suncheon 57922, Jeonnam, Republic of Korea; gmor2224@naver.com; 4 Department of Natural Cosmetics Science, Graduate School, Sunchon National University, 255 Jungangno, Suncheon 57922, Jeonnam, Republic of Korea; thelsg@scnu.ac.kr; 5 Glocal University Project Team, Sunchon National University, 255 Jungangno, Suncheon 57922, Jeonnam, Republic of Korea; 6 Department of Biosciences, Mokpo National University, Muan 58554, Jeonnam, Republic of Korea

**Keywords:** *M. oleifera*, PPARγ, adiponectin, FABP4, molecular docking, obesity, diabetes

## Abstract

**Background/Objectives**: *Moringa oleifera* is a matrix plant with the high potential to cure several diseases with its medicinal and ethnopharmacological value and nutraceutical properties. In this study, we investigated the chemical and biological properties of this plant cultivated in our local region. **Methods**: Leaves, roots, seeds, stem bark, and twigs of *oleifera* were extracted and evaluated bioactivities targeting intracellular lipid accumulation and adipocyte differentiation in 3T3-L1 preadipocytes, and UHPLC-ESI-Orbitrap-MS/MS-Based molecular networking guided isolation and dereplication of metabolites from these extracts. **Results**: Five extracts of different organs of *M*. *oleifera* significantly stimulated intracellular lipid accumulation and adipocyte differentiation in 3T3-L1 preadipocytes in a concentration-dependent manner. These extracts markedly increased the expression of genes related to adipogenesis and lipogenesis. Notably, these extracts promoted peroxisome proliferator-activated receptor γ (PPARγ) activity and the expression of its target genes, including phosphoenolpyruvate carboxykinase, fatty acid-binding protein 4, and perilipin-2. These adipogenic and lipogenic effects of Moringa extracts through the regulation of PPARγ activity suggests their potential efficacy in preventing or treating type 2 diabetes. Furthermore, chemical investigation revealed high contents of phytonutrients as rich sources of secondary metabolites including glycosides, flavones, fatty acids, phenolics, and other compounds. In addition, in silico studies on major components of these extracts revealed the bioavailability of major components through their binding affinity to respective proteins targeting adipocyte differentiation.

## 1. Introduction

*Moringa oleifera* Lam. belongs to the Moringaceae family. This plant is widely distributed in sub-tropical and tropical areas over the world [1]. It is also known as a food commodity containing an enormous amount of natural nutrients. Among the organs of this plant, the leaves, fruits, and flowers are consumed as nutritive vegetables in many countries [2,3]. Previous studies have reported that *M*. *oleifera* has a variety of potent pharmacological effects such as antioxidant, anti-inflammatory, anticancer, antihypertensive, and cholesterol-lowering effects [4]. These strong bioactivities of this plant might be due to its high contents of phytochemicals such as amino acids, proteins, vitamins, polyphenols, flavonoids, alkaloids, and cardiac glycosides [5]. This plant can grow in a variety of harsh environmental conditions such as in high temperatures and in areas with limited water availability [6]. Due to its high nutritional potential, *M*. *oleifera* is cultivated in many regions over the world, including our local region. The chemical investigation of its metabolites was performed through the application of a dereplication technique using liquid chromatography–mass spectrometry (LC-MS) known to provide a high sensitivity and effectiveness for identifying natural products [7].

Obesity is a major contributor to metabolic diseases such as diabetes, hypertension, and hyperlipidemia, posing a threat to global public health. Globally, the prevalence of obesity has been rising gradually, resulting in substantial health and economic burdens [8]. Adipose tissue is not only an energy storage depot [9] but also an active endocrine organ that plays crucial roles in energy homeostasis, hormone secretion, and temperature regulation [10]. Adipose tissue secretes a variety of bioactive molecules called adipocytokines such as adiponectin, leptin, and others [11]. Among them, adiponectin is known as an important adipocytokine essential for numerous signal pathways, enhancing insulin sensitivity, reducing inflammatory effects, and contributing to the regulation of glucose and lipid metabolism [12]. Obesity causes a decrease in the secretion of beneficial adipocytokines such as adiponectin, which can increase the production of pro-inflammatory cytokines and tumor necrosis factor α. This imbalance in adipocytokines contributes to the development of insulin resistance and various metabolic disorders, including type 2 diabetes, cardiovascular diseases, and certain cancers [11,12,13,14]. Our growing understanding of biology has highlighted the importance of adipose tissue in metabolic health and disease, making it a crucial target for therapeutic therapies to lessen the negative consequences of obesity.

Adipocyte development is significantly influenced by transcriptional factors such as PPARγ and CCAAT/enhancer-binding proteins (C/EBPs), as well as hormonal signaling pathways such as insulin and glucocorticoids [14,15]. During adipogenesis, C/EBPβ is rapidly induced by hormonal signals and acts as a transcriptional regulator of PPARγ [16,17]. PPARγ forms a heterodimer with retinoid x receptor α (RXRα) and binds to specific DNA sequences known as PPRE in target gene promoters to promote the transcription of genes involved in adipocyte maturation [15]. Agonists of PPARγ, such as thiazolidinediones (e.g., pioglitazone, rosiglitazone), enhance insulin sensitivity and regulate glucose metabolism, making them effective treatments for type 2 diabetes. These agonists promote adipocyte differentiation, reduce circulating free fatty acids, and increase adiponectin levels, which collectively improve insulin sensitivity and lower blood glucose. However, side effects such as weight gain and fluid retention limit their use, although they remain important for managing insulin resistance in type 2 diabetes [18].

In this study, we investigated the biological efficacy of different extracts of *M*. *oleifera* organs on adipocyte differentiation and PPARγ activity and explored mechanisms involved. A method utilizing LC-MS was developed to identify chemicals derived from various extracts by the dereplication strategy using mass fragmentation and isotope patterns in collaboration with a public mass data bank or comparison with those of standards to investigate secondary metabolites from five organ extracts of this plant. Furthermore, major components from the most active extract were predicted based on their binding affinity with targeted proteins using molecular docking analysis.

## 2. Results and Discussion

### 2.1. Moringa Leaf, Stem Bark, and Twig Extracts Stimulate Adipocyte Differentiation

**Figure 1:**
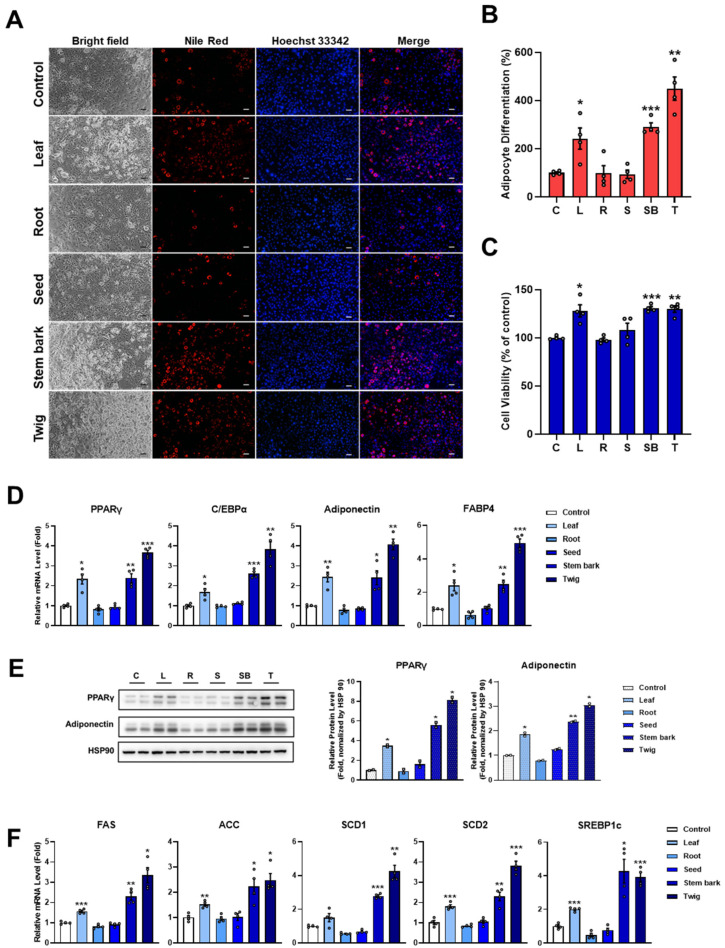
Stimulatory effects of Moringa extracts on adipocyte differentiation. The 3T3-L1 preadipocytes were cultured in a 0.1× differentiation induction medium for 6 days, either without (control) or with each Moringa extracts (100 μg/mL). (**A**) Cells were differentiated for 6 days were then stained with Nile Red to visualize lipid droplets and Hoechst 33342 to stain nuclei. Images were acquired by epifluorescence microscopy. Scale bar = 100 µm. (**B**,**C**) Quantification of Nile Red intensity and cell viability was performed using ImageJ software(version 1.54f). Nile Red fluorescence was normalized to the cell count determined by Hoechst 33342 staining. (**D**) mRNA expression levels of adipogenic genes were measured by qRT-PCR analysis. Data are shown as mean ± SEM (*n* = 4 per group). (**E**) The protein expression levels of PPARγ and adiponectin were analyzed using Western blotting. Results are shown as mean ± SEM (*n* = 2 per group). (**F**) mRNA expression levels of lipogenic genes were measured by qRT-PCR. Relative mRNA expression levels were normalized to 36B4 and are shown as mean ± SEM (*n* = 4 per group). All data are indicated as *, *p* < 0.05; **, *p* < 0.01; ***, *p* < 0.001 versus control group.

To determine the effects of Moringa extracts on adipocyte differentiation, 3T3-L1 preadipocytes were used. Adipocyte differentiation induced by Moringa extracts from various regions at a concentration of 100 µg/mL in 3T3-L1 preadipocytes cultured under differentiation-inducing conditions for 6 days, was assessed by Nile Red fluorescence staining. As illustrated in Figure 1A, only leaf, stem bark, and twig extracts increased intracellular lipid accumulation, a hallmark of adipocyte differentiation, without cytotoxic effects as compared to the control group (Figure 1A–C). Leaf, stem bark, and twig extracts of Moringa increased adipocyte differentiation by 241.91 ± 43.97%, 290.31 ± 16.88%, and 449.71 ± 48.19%, respectively (Figure 1B). However, neither Moringa root nor seed extract could promote adipocyte differentiation. To investigate the molecular mechanisms involved in the regulation of adipocyte differentiation by Moringa extracts, we measured the expression levels of adipogenic and lipogenic genes at both mRNA and protein levels using quantitative real-time PCR (qRT-PCR) and Western blotting analysis, respectively.

Results showed that mRNA levels of several adipogenic genes (PPARγ, C/EBPα, adiponectin, and FABP4) were decreased by leaf, stem bark, and twig extracts of Moringa (Figure 1D). Consistent with qRT-PCR results, protein levels of PPARγ and adiponectin were upregulated by these three extracts (Figure 1E). qRT-PCR analysis also revealed upregulated expression levels of lipogenic genes such as FAS, ACC, SCD1, SCD2, and SREBP1c (Figure 1F), suggesting that these extracts could enhance adipocyte differentiation and function. The upregulation of lipogenic genes such as FAS, SCD1, SCD2, ACC, and SREBP1c during adipocyte differentiation is crucial for lipid metabolism and adipocyte function. FAS and ACC are key enzymes in fatty acid synthesis, essential for lipid storage and energy balance, while SCD1 and SCD2 regulate fatty acid desaturation and maintain membrane composition. SREBP1c, as a master regulator, promotes the expression of these genes, ensuring proper lipid accumulation and adipocyte maturation [19]. This upregulation reflects an active lipid biosynthesis pathway essential for adipocyte development and metabolic regulation.

The inhibitory effects of Moringa extracts on adipocyte differentiation were also examined (Figure S1, Supplementary Materials). Overall, Moringa extracts did not suppress intracellular lipid accumulation, or the expression of genes associated with adipocyte differentiation or fatty acid synthesis during the differentiation process compared to the control group. Hence, subsequent analyses focused on the promotion of adipocyte differentiation by leaf, stem bark, and twig extracts.

### 2.2. Leaf Extract of M. oleifera Stimulates Adipocyte Differentiation

**Figure 2:**
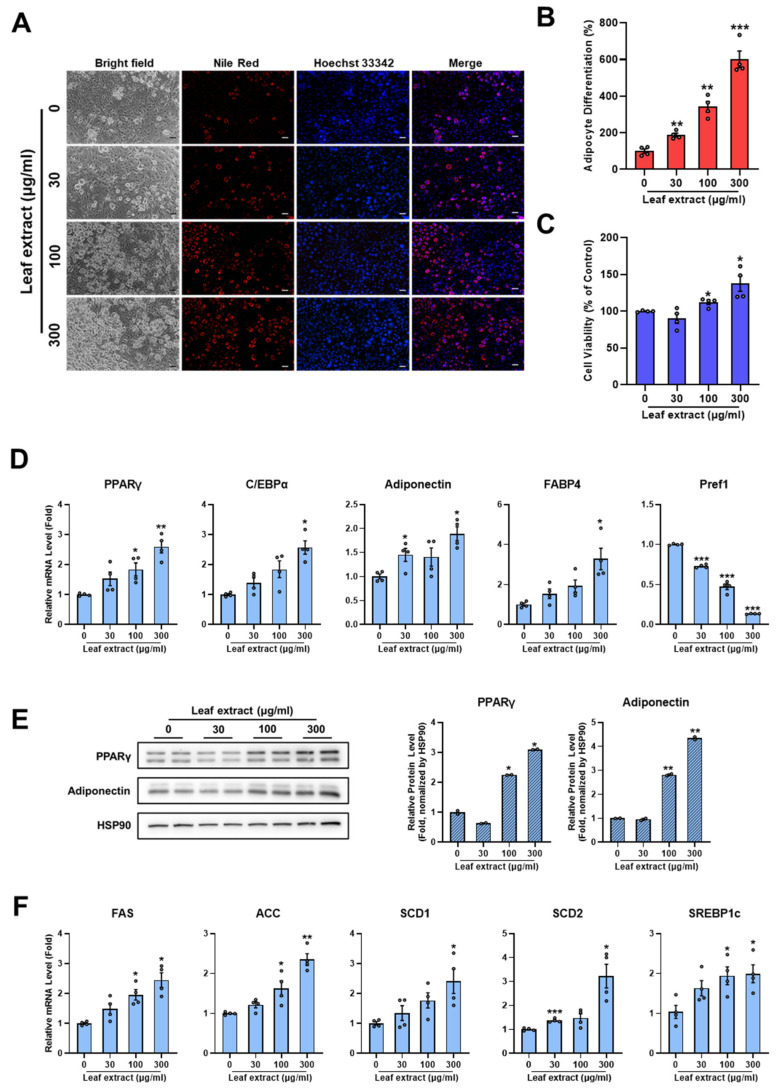
Leaf extract of *M. oleifera* promotes adipocyte differentiation. The 3T3-L1 preadipocytes were cultured in a 0.1× differentiation induction medium for 6 days, either without (control) or with various concentrations of leaf extract (30, 100, and 300 μg/mL) of *M. oleifera*. (**A**) Following 6 days of differentiation, cells were stained with Nile Red and Hoechst 33342. Images were acquired by epifluorescence microscopy. Scale bar = 100 µm. (**B**,**C**) ImageJ software was used to quantify Nile Red staining intensity and cell viability. Data are shown as mean ± SEM (*n* = 4 per group). (**D**) mRNA expression levels of adipogenic genes were analyzed using qRT-PCR analysis. (**E**) Protein levels of PPARγ and Adiponectin were determined by Western blotting. Results are shown as mean ± SEM (*n* = 2 per group). (**F**) mRNA expression levels of lipogenic genes were measured by qRT-PCR analysis. Relative mRNA expression levels were normalized to 36B4 and are shown as mean ± SEM (*n* = 4 per group). All data are indicated as *, *p* < 0.05; **, *p* < 0.01; ***, *p* < 0.001 versus control group.

To further confirm the concentration-dependent effects of leaf extract of *M. oleifera* on adipocyte differentiation, various concentrations (30–300 µg/mL) were tested during the differentiation process. The leaf extract effectively increased intracellular lipid accumulation in a dose-dependent manner, with no observed cytotoxicity at these concentrations (Figure 2A–C). Subsequent analyses of mRNA and protein expression were conducted to elucidate the mechanisms by which the leaf extract promotes adipocyte differentiation. The findings revealed that genes involved in adipocyte differentiation, including PPARγ, C/EBPα, adiponectin, and FABP4, were significantly upregulated by the leaf extract (Figure 2D). Adipogenic transcription factors PPARγ and C/EBPα were increased by 2.59 ± 0.20-fold and 2.56 ± 0.22-fold, respectively, at a concentration of 300 µg/mL. Conversely, Pref-1 gene expression was significantly reduced by *M. oleifera* leaf extract in a concentration-dependent manner. Additionally, protein levels of PPARγ and adiponectin were also elevated by 3.10 ± 0.01-fold and 4.34 ± 0.05-fold, respectively, at a concentration of 300 µg/mL (Figure 2E). Furthermore, genes related to fatty acid synthesis, including FAS, ACC, SCD1, SCD2, and SREBP1c, showed a concentration-dependent increase through the treatment of Moringa leaf extract (Figure 2F). These results suggest that *M. oleifera* leaf extract clearly promotes adipocyte differentiation by regulating lipid accumulation and the expression of adipogenic and lipogenic genes. In contrast to our findings, several studies have demonstrated the anti-adipogenic properties of *M. oleifera* leaf extract [20,21].

### 2.3. Stem Bark Extract of M. oleifera Induces Adipocyte Differentiation

**Figure 3:**
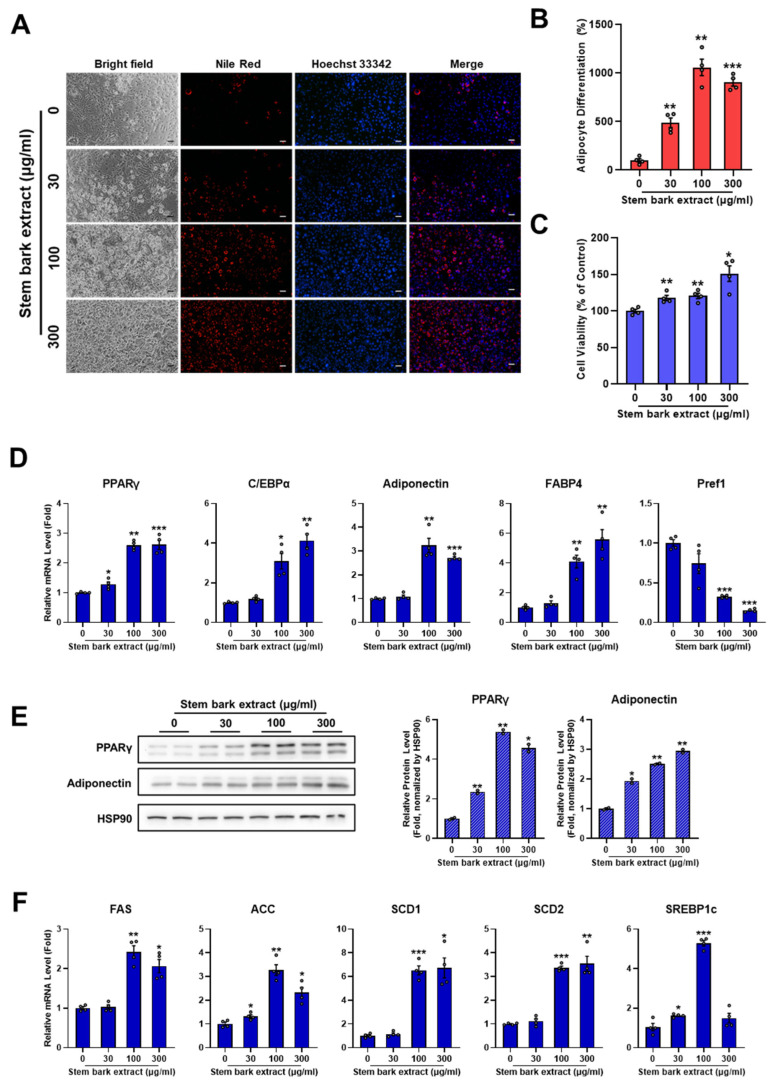
Stem bark extract of *M. oleifera* induced adipocyte differentiation. The 3T3-L1 preadipocytes were cultured in a 0.1× differentiation induction medium for 6 days, either without (control) or with various concentrations of the stem bark extract (30, 100, and 300 μg/mL). (**A**) After 6 days, cells were stained with Nile Red and Hoechst33342. Images were acquired by epifluorescence microscopy. Scale bar = 100 µm. (**B**,**C**) Quantification of Nile Red intensity and cell viability was performed using ImageJ software. Data are shown as mean ± SEM (*n* = 4 per group). (**D**) mRNA expression levels of adipogenic genes were measured using qRT-PCR analysis. (**E**) Protein expression levels of PPARγ and adiponectin were assessed by Western blotting, with results shown as mean ± SEM (*n* = 2 per group). (**F**) mRNA expression levels of lipogenic genes were measured using qRT-PCR analysis. Relative mRNA expression levels were normalized to 36B4 and are shown as mean ± SEM (*n* = 4 per group). All data are presented as *, *p* < 0.05; **, *p* < 0.01; ***, *p* < 0.001 versus control group.

Using a similar approach to previous experiments, we examined concentration-dependent effects of stem bark extract of *M. oleifera* on adipocyte differentiation. As shown in Figure 3A–C, stem bark extract enhanced intracellular lipid accumulation in a dose-dependent manner without showing any cytotoxicity. The analysis of Nile Red staining revealed that adipocyte differentiation was increased by approximately 488.68 ± 49.58%, 1058.13 ± 84.92%, and 904.80 ± 39.26% by stem bark extract at 30, 100, and 300 µg/mL, respectively (Figure 3B). Gene and protein expression level changes induced by stem bark extract of *M. oleifera* were analyzed using qPCR and Western blotting analysis. Results showed that stem bark extract significantly elevated the expression of adipogenic genes in a concentration-dependent manner. Specifically, transcription factors PPARγ and C/EBPα known to govern adipocyte differentiation were increased by 2.59 ± 0.08-fold and 3.09 ± 0.39-fold, respectively, by the stem bark extract (100 µg/mL) of *M. oleifera*. Adipocyte marker genes adiponectin and FABP4 also showed increases of 3.24 ± 0.30-fold and 4.10 ± 0.43-fold, respectively, after treatment with stem bark extract at a concentration of 100 µg/mL. Conversely, Pref-1 gene expression was markedly reduced by stem bark extract of *M. oleifera*. PPARγ and adiponectin protein levels were upregulated by the stem bark extract of *M. oleifera* in a dose-dependent manner. They were increased 5.39 ± 0.10-fold and 2.51 ± 0.03-fold by stem bark extract at a concentration of 100 µg/mL. Fatty acid synthesis-related genes were also upregulated by the stem bark extract of *M. oleifera* in a concentration-dependent manner, with expression levels of FAS, ACC, SCD1, SCD2, and SREBP1c genes increased by 2.43 ± 0.16-fold, 3.28 ± 0.23-fold, 6.52 ± 0.38-fold, 3.37 ± 0.07-fold, and 5.29 ± 0.14-fold, respectively, after treatment with stem bark extract of *M. oleifera* at a concentration of 100 µg/mL. These data indicate that stem bark extract of *M. oleifera* can enhance adipocyte differentiation by activating intracellular lipid accumulation and the expression of genes related to adipogenesis and lipogenesis.

### 2.4. Twig Extract of M. oleifera Increases Adipocyte Differentiation

**Figure 4:**
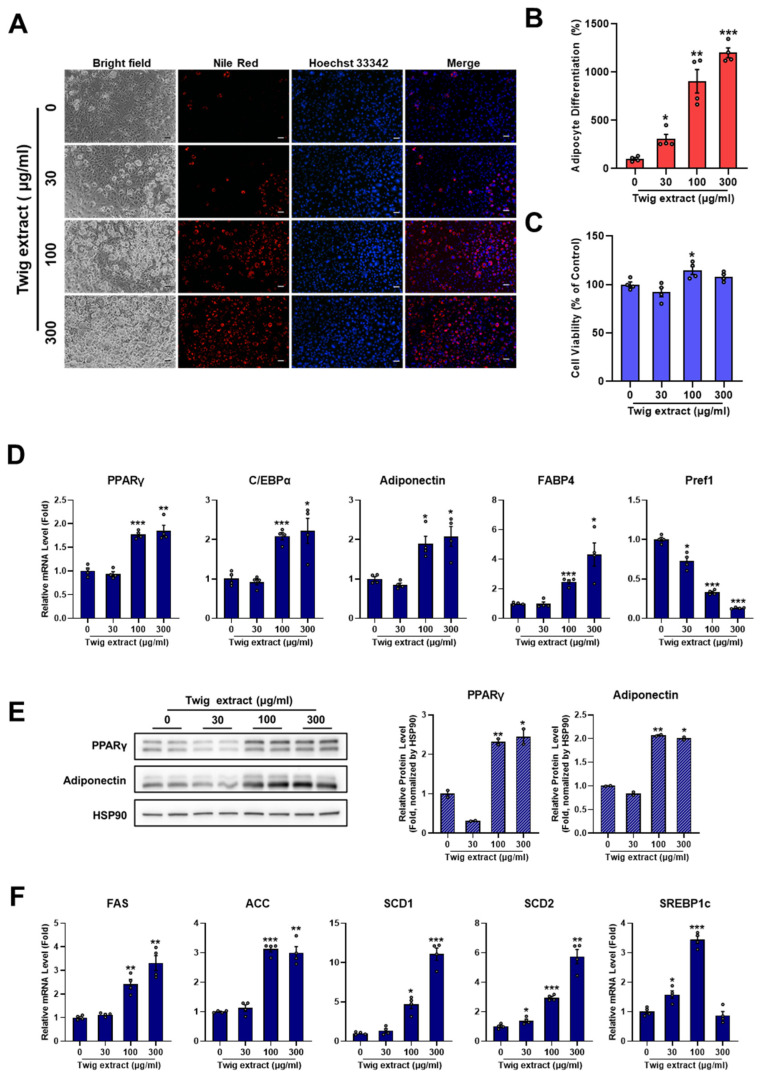
Twig extract of *M. oleifera* stimulates the adipocyte differentiation of 3T3-L1 preadipocytes. Cells were cultured in a 0.1× differentiation induction medium for 6 days, either without (control) or with various concentrations of MRG twig extracts. (**A**) Cells differentiated for 6 days were stained with Nile Red and Hoechst33342. Images were acquired by epifluorescence microscopy. Scale bar = 100 µm. (**B**,**C**) The intensity of Nile Red staining and cell viability were quantified using ImageJ software. Results are shown as mean ± SEM (*n* = 4 per group). (**D**) mRNA expression levels of adipogenic genes were analyzed using qRT-PCR analysis. (**E**) Expression levels of PPARγ and adiponectin proteins were determined by Western blotting, with data shown as mean ± SEM (*n* = 2 per group). (**F**) mRNA expression levels of lipogenic genes were measured using qRT-PCR analysis. Relative mRNA levels were normalized to 36B4 and are presented as mean ± SEM (*n* = 4 per group). Statistical significance is indicated as *, *p* < 0.05; **, *p* < 0.01; ***, *p* < 0.001 versus control group.

Similarly, we investigated concentration-dependent effects of twig extract of *M. oleifera* on adipocyte differentiation using the same method. As shown in Figure 4A, twig extract promoted intracellular lipid accumulation in a dose-dependent manner without causing cytotoxicity. Adipocyte differentiation assessed by Nile Red staining was increased by 306.30 ± 48.14%, 906.45 ± 121.03%, and 1200.61 ± 51.57% at 30, 100, and 300 µg/mL, respectively. No cytotoxic effects were observed in any treatment groups. Changes in mRNA and protein expression levels induced by twig extract of *M. oleifera* were confirmed through qPCR and Western blot analyses, respectively. Results indicated that twig extract of *M. oleifera* significantly enhanced the expression of adipogenic genes in a concentration-dependent manner. In particular, the expression levels of PPARγ and C/EBPα transcription factors regulating adipocyte differentiation were increased by 1.77 ± 0.04-fold and 2.08 ± 0.09-fold, respectively, by the twig extract (100 µg/mL) of *M. oleifera*, while expression levels of adiponectin and FABP4 were increased by 1.89 ± 0.20-fold and 2.47 ± 0.14-fold, respectively. However, Pref-1 gene expression was notably decreased by twig extract of *M. oleifera*. Additionally, at the protein level, PPARγ and adiponectin were upregulated by twig extract of *M. oleifera* in a concentration-dependent manner. They were increased 2.32 ± 0.07-fold and 2.07 ± 0.02-fold, respectively, by twig extract at 100 µg/mL. Genes related to fatty acid synthesis were also upregulated by twig extract of *M. oleifera* in a dose-dependent manner. The expression levels of FAS, ACC, SCD1, SCD2, and SREBP1c were increased 2.42 ± 0.36-fold, 3.13 ± 0.56-fold, 4.67 ± 0.73-fold, 2.96 ± 0.36-fold, and 3.44 ± 0.58-fold, respectively, by Moringa twig extract at 100 µg/mL. Overall, these observations demonstrate that twig extract of *M. oleifera* has markedly adipogenic effects on adipocyte differentiation (Figure 4A–F). There have been no previous studies on the efficacy of *M. oleifera* twigs in regulating adipocyte differentiation. However, some studies reported that two fractions from *M. oleifera* twigs exhibit antioxidant, antimicrobial, and xanthine oxidase inhibitory activities, suggesting potential as antihyperuricemic and antimicrobial agents [22].

### 2.5. Leaf, Stem Bark, and Twig Extracts of M. oleifera Enhance PPARγ Activity and the Expression of PPARγ Target Genes

**Figure 5:**
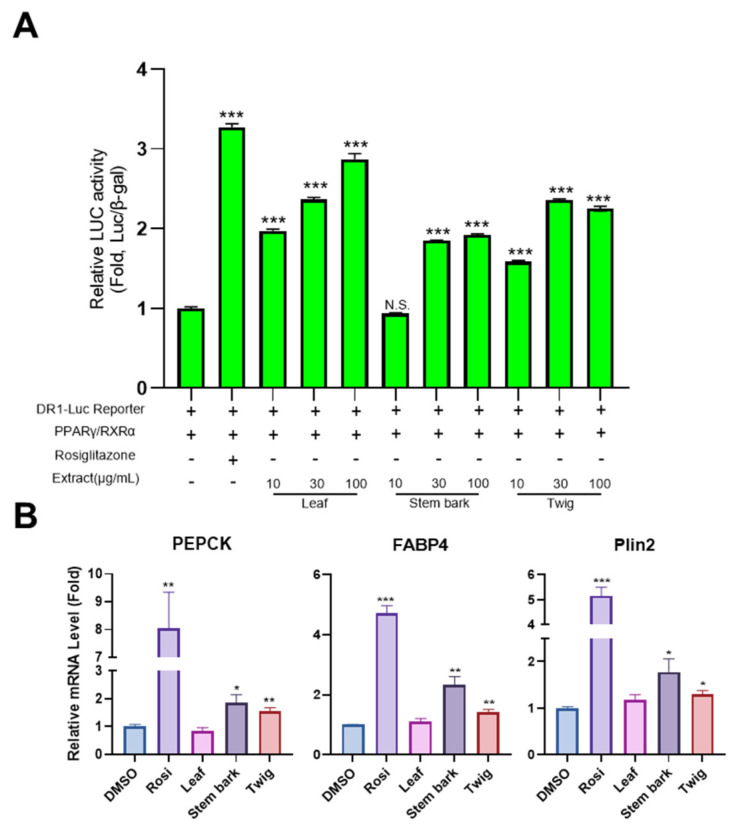
The leaf, stem bark, and twig extracts of *M. oleifera* increase PPARγ activity and the expression of PPARγ target genes. (**A**) h293a cells were transfected with DR-1-Luc, which contained three PPRE sequences upstream of the luciferase gene and indicated PPARγ and RXRα. After a day, indicated Moringa extracts of various doses (10, 30, and 100 μg/mL) or 10 μM of rosiglitazone were treated for a day. Measured luminescence levels were normalized with O.D values of the *β*-gal assay. N.S., not significant. (**B**) Differentiated adipocytes were seeded in a 12-well plate and treated with 100 μg/mL of indicated Moringa extracts or 10 μM rosiglitazone (positive control) or DMSO (negative group) for 2 days. Each RNA was used for qRT-PCR analysis. All data are presented as the mean ± SEM of two independent experiments (*n* = 4). *, *p* < 0.05; **, *p* < 0.01; ***, *p* < 0.001 versus control group.

PPARγ is a crucial transcription factor in adipocyte differentiation. To assess whether leaf, stem bark, and twig extracts of *M. oleifera* could regulate PPARγ activity, a luciferase reporter assay was performed using a PPRE-inserted reporter capable of binding to PPARγ/RXRα heterodimer. After transfecting HEK293 cells with the reporter and PPARγ/RXRα, cells were treated with Moringa extract or rosiglitazone for 24 h, followed by luciferase analysis. As shown in Figure 5A, rosiglitazone, a PPARγ agonist used as a positive control, enhanced luciferase activity. Interestingly, Moringa extracts also increased the luciferase activity in a concentration-dependent manner. This indicates that Moringa extracts can directly regulate PPARγ activity. Additionally, to determine if Moringa extract could directly promote PPARγ activity, we examined the expression of PPARγ target genes such as PEPCK, FABP4, and Plin2 following treatment with Moringa extract in differentiated adipocytes (Figure 5B). Results showed that both stem bark and twig extracts of *M. oleifera* significantly upregulated PPARγ target genes including PEPCK, FABP4, and Plin2, while the leaf extract of Moringa tended to increase Plin2 gene expression. These findings suggest that Moringa extracts are likely to promote adipocyte differentiation by enhancing PPARγ activity.

### 2.6. Identification of Metabolites from Five Organs of M. oleifera

**Figure 6:**
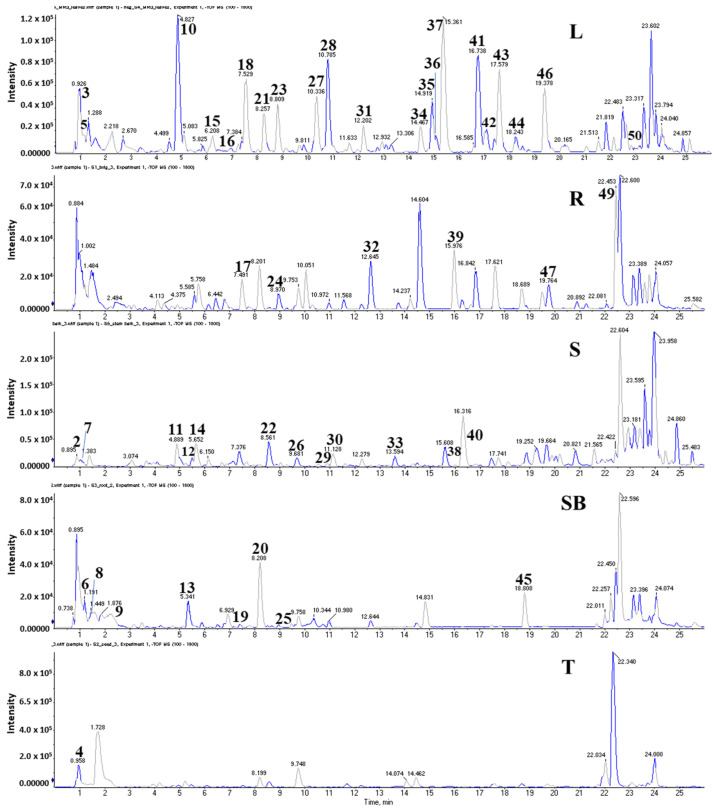
Chromatograms of five extracts of leaves (L), roots (R), seeds (S), stem bark (SB), and twigs (T) of *M*. *oleifera*.

**Figure 7:**
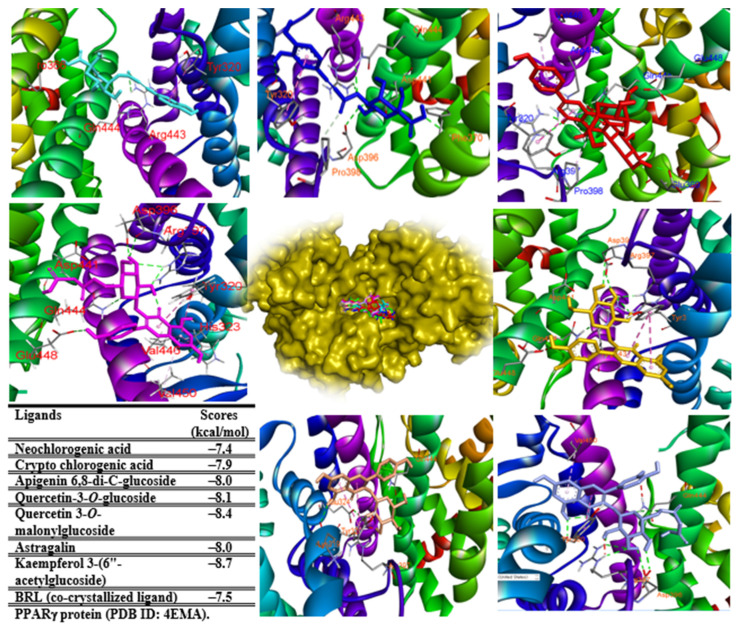
Molecular docking of compounds **10** (cyan), **18** (blue), **28** (red), **37** (light orange), **41** (light blue), **43** (yellow), **46** (magenta), and co-crystallized ligand (2.4-thiazolidinedione, 5-[[4-[2-(methyl-2pyridinylamino)ethoxy]phenyl]methyl]-9cl, docked into PPARγ protein (PDB ID 4EMA).

To investigate secondary metabolites of *M*. *oleifera*, a cultured plant, we employed a dereplication technique using an LC-MS/MS system (Figure 6). As a result, chemicals were identified from extracts of different organs of M. oleifera by accessing high mass precursors, MS2 fragmentation patterns, and comparing them to those reported in public databases (Figure 7) such as Massbank (http://www.massbank.jp/Search, accessed 19 August 2024), Human Metabolom Database (http://www.hmdb.ca, 21 August 2024), GNPS (https://www.mzcloud.org, accessed 16 August 2024), and Metlin (https://metlin.scripps.edu, accessed 13 August 2024).

#### 2.6.1. Glycosides

Peaks **1** and **5** generated similar product ions at *m*/*z* 179.0563 [M-H]^-^ and 179.0568 [M-H-Fruc]^-^ corresponding to glucose and sucrose [23], respectively. Peak **2** showed a parent ion at *m*/*z* 341.1089 [M-H]^-^. Spectrum of peak **2** displayed a parent ion at *m*/*z* 181.0696 [M-H]^-^ and other product ions at *m*/*z* 163.0590 [M-H-H_2_O]^-^, 119.0375 [M-H-H_2_O-C_2_H_5_O]^-^, and 101.0255 [M-H-H_2_O-C_2_H_5_O-H_2_O]^-^. Peak **2** was identified as mannitol [24]. Peak **21** showed a precursor ion at *m*/*z* 612.1061 [M-H]^-^ corresponding to acetyl-4-(*α*-L-rhamnopyranosyloxy)benzyl glucosinolate previously identified from *M*. *oleifera* [25]. Peak **24** displayed a parent ion at *m*/*z* 447.1503 and generated fragments at *m*/*z* 401.1465 [M-H_2_O]^-^ and 269.1031 [M-H_2_O-OGlc]^-^ by losing water and glucose units. Thus, peak **24** was determined as anacardoside. Peak **28** showed parent ions at *m*/*z* 324.1087 [M-H]^-^ and 278.1050 [M-H_2_O-CHO]^-^. It was identified as 2-formylphenyl 2-acetamido-2-deoxy-*β*-D-glucopyranoside (Table 1).

#### 2.6.2. Flavones

A total of 11 flavonoid derivatives were identified in Moringa extracts, as shown in Table 1. The fragmentation pathways of flavones and their glycosides have been well studied [26,27]. Ion fragmentations were characterized by retro-Diels–Alder (RDA) cleavage of the C-ring and multiple neutral loss pathways, whereas the sequential elimination of sugar residues, combined with aglycone fragments, was indicative of flavonoids. According to chemical structural formula and MS/MS fragmentation, flavones could be classified as flavone-*O*-glycosides and flavone-*C*-glycosides. Peaks **29**, **35**, and **38** showed fragment ions at *m*/*z* 149.0228 [C_7_H_3_O_4_]^-^ and 117.0371 [C_8_H_5_O]^-^ corresponding to the ^1,3^A^-^ and ^1,3^B^-^ rings, respectively, if C-ring cleavage produced complementary fragment ions [28] of the A- and B-rings. At the same time, fragment ion at *m*/*z* 311.0569 [M-2H-C_8_H_5_O]^-^ was also observed by loss of the ^1,3^B^-^ ring ion. On the other hand, its fragmentation pathway may produce an ion at *m*/*z* 283.0611 [M-2H-C_8_H_5_O-CO]^-^ by losing the ^1,3^B^-^ ring and C=O ions. Subsequently, it might produce an ion at *m*/*z* 269.0462 [M-H-C_8_H_5_O-CO-H_2_O]^-^ by losing a water molecule.

The precursors of **29**, **35**, and **38** peaks were also detected at *m*/*z* 593.1509 (**29**), 431.0982 (**35**), and 431.0985 (**38**) corresponding to apigenin-6,8-di-*C*-glucoside (**29**), vitexin (**35**), and isovitexin (**38**), respectively, based on public mass data bank (Table 1). These chemicals belong to flavone-*C*-glycosides. Peaks **37**, **39**, **41**, and **43**–**49** displayed fragmentation pathway of flavone-*O*-glycosides due to their cleavages of glycosides showing fragment ions of aglycone at *m*/*z* 300.0277 [Quercetin-H]^-^ and *m*/*z* 314.0447 [Isorhamnetin-H]^-^. Its mass spectrum also displayed a product ion at *m*/*z* 178.9983 [C_6_H_10_O_6_]^-^, showing fragments of glucose or galactose units [29]. Their mother ions were observed at *m*/*z* 609.1467 [M-H]^-^, 463.0886 [M-H]^-^, 549.0891 [M-H]^-^, 505.0985 [M-H]^-^, and 549.0891 [M-H]^-^, corresponding to rutin (**37**), quercetin-3-*O*-glucoside (**39**), quercetin 3-(6″-acetylglucoside) (**41**), and quercetin 3-*O*-malonylglucoside (**43**), respectively, based on reference standard and a public mass data bank (Table 1). Peaks **45**–**47** and **49** showed precursor ions peaks at *m*/*z* 593.1516 [M-H]^-^, 447.0930 [M-H]^-^, and 489.1041 [M-H]^-^. On the other hand, they might produce fragment ions by losing sugar units at *m*/*z* 284.0337 (**45**) [M-H-Glc-Rha]^-^, 284.0328 (**46**) [M-H-Glc]^-^, and 284.0330 (**49**) [M-H-Glc-Ac]^-^. At the same time, this aglycone may undergo RDA cleavage to produce product ions at *m*/*z* 255.0302 [Kaemferol-H-CO]^-^ and 227.0353 [Kaemferol-H-C_2_O_3_]^-^ of kaemferol glycone [30,31]. Therefore, these peaks were identified as kaempferol-3-rutinoside (**45**), astragalin (**46**), and kaempferol 3-(6″-acetylglucoside) [25] by comparing them with standards or previous reports.

#### 2.6.3. Fatty Acid

Peaks **31**, **34**, **40**, **42**, **51**, and **53** were identified as fatty acid derivatives due to their fragment ions produced from mass spectra. Briefly, peak **31** showed ions peaks at *m*/*z* 173.0808 [M-H]^-^, 155.0713 [M-H_2_O]^-^, 129.0931 [M-H-COOH]^-^, and 111.0821 [M-H_2_O-COOH]^-^ by losing water and COOH groups from its molecular structure. This peak was established as suberic acid by analyzing mass data from experimental and public data banks. Peak **34** showed a precursor ion peak at *m*/*z* 177.1024 [M-H]^-^ and further ionization with product ions at *m*/*z* 155.1078 [M-H_2_O]^-^ and 127.1125 [M-H-COOH]^-^. This peak was tentatively identified as 9-oxo-nonanoic acid [32]. The mass spectrum of peak **40** produced ion peaks at *m*/*z* 261.1341 [M-H]^-^ and 187.0967 [M-H-Glyceryl]^-^. This peak was identified as 9-(2,3-dihydroxypropoxy)-9-oxononanoic acid based on a public mass data bank. Peak **42** displayed a deprotonated ion at *m*/*z* 187.0977 [M-H]^-^. Other product ions at *m*/*z* 169.0869 [M-H_2_O]^-^, 143.1088 [M-H-COOH]^-^, and 125.0975 [M-H-COOH-C_2_H_2_]^-^ were also produced. Peak **42** was tentatively identified as azelaic acid. Peak **51** showed a precursor ion at *m*/*z* 201.1129 [M-H]^-^ with other product ions at *m*/*z* 183.1023 [M-H_2_O]^-^ and 139.1130 [M-H_2_O-COOH]^-^. Peak **51** was identified as decanedioic acid based on the public data bank. The mass spectrum of peak **53** showed a fragment at *m*/*z* 229.1435 with a loss of 97 Da (C_7_H_13_) from its parent ion at *m*/*z* 327.2171 [M-H]^-^. Further ionization generated another fragment at *m*/*z* 211.1332 [M-H_2_O-C_7_H_13_]^-^. Therefore, peak **51** was identified as 9,12,13-trihydroxy-10,15-octadecadienoic acid.

#### 2.6.4. Phenolics

The mass spectra of peaks **3**, **10**, **15**, **16**, and **18** showed a similar product ion at *m*/*z* of about 191 [Quinic acid-H]^-^. Peaks **3**, **10**, **16**, and **18** also displayed another similar product ion at *m*/*z* about 179 [Caffeoyl] which was produced from parent ions about *m*/*z* 353 by losing a caffeoyl unit. Peaks **3**, **10**, **16**, and **18** were identified as quinic acid (**3**), neochlorogenic (**10**), chlorogenic (**6**), and cyptochlorogenic acid (**18**), respectively. The mass spectrum of peak **15** showed a parent ion at *m*/*z* 337.0931 [M-H]^-^. Its fragmentation generated another product ion at *m*/*z* 163.0404 [*p*-Courmaroyl-H]^-^ by losing 191.0567 [Quinic acid-H]^-^. Therefore, peak **15** was identified as *p*-courmaroylquinic acid. Peak **27** showed a parent ion at *m*/*z* 165.0552 [M-H]^-^ and produced other fragments at *m*/*z* 147.0445 [M-H_2_O]^-^, 119.0503 [M-H_2_O-CO]^-^, and 103.0558 [M-H_2_O-OH-CO]^-^. Thus, peak **27** was determined as 4-hydroxyhydrocinnamic acid. Peak 48 displayed a precursor ion at *m*/*z* 361.1659 [M-H]^-^. Its fragmentation pathway generated product ions at *m*/*z* 346.1438 [M-H]^-^ by losing a water atom. Thus, peak **48** was identified as secoisolariciresinol by interpreting mass information both from a reference [33] and a public data bank (Table 1).

#### 2.6.5. Other Compounds

Peak **6** showed parent ion at *m*/*z* 191.0186 [M-H]^-^ and product ions at *m*/*z* 173.0086 and 111.0103 corresponding to reduction in a water atom and COOH groups, respectively. Peak **6** was identified as citric acid [34]. Peak **13** revealed a parent ion at *m*/*z* 175.0615 [M-H]^-^. Its MS^2^ fragmentation generated ions at *m*/*z* 157.0590 and 115.0414 due to loss of water (*m*/*z* 18) and loss of acetic acid, respectively. Therefore, peak **13** was identified as 2-isopropylmalic acid [35]. Peak **32** displayed parent ion at *m*/*z* 439.1818 [M-H]^-^. Its MS^2^ fragments showed ions at *m*/*z* 261.1341 [M-H-Glc]^-^ and 179.0559 [Glc]^-^. Peak **32** was identified as nonioside D by analyzing its mass spectrum and comparing it with a previous report [36]. In addition, peaks **7**, **8**, **12**, **17**, **26**, and **30** were identified using public mass data bank. Peaks **4**, **11**, **14**, **19**, **20**, **23**, **33**, and **50** were unknown metabolites until current study.

### 2.7. In Silico Study

PPARγ, FABP4, and adiponectin proteins play crucial roles in the regulation of adipocyte differentiation and function, making them important targets for the prevention and treatment of metabolic diseases. PPARγ promotes adipocyte differentiation and enhances fatty acid synthesis and storage, thereby increasing insulin sensitivity. FABP4 is primarily expressed in adipocytes and macrophages. It binds and transports fatty acids, contributing to fatty acid metabolism and energy balance regulation. Adiponectin is secreted mainly by adipocytes. It enhances insulin sensitivity to regulate blood glucose levels and promotes fatty acid oxidation to increase energy expenditure.

Previous studies demonstrated that neochlorogenic acid may improve kidney function and reduce fibrosis. Therefore, this compound may regulate glucolipotoxicity-induced diabetic nephropathy by targeting lipid metabolism and modulated JAK-STAT, pAKT, Ras, and NF-κB signaling pathways [37]. Cryptochlorogenic acid exhibited many abilities to reduce blood pressure in diabetes and showed the protective effect on impairment of pancreases function of rat with diabetes [38]. Pantothenic acid dose-dependently increased the expression of FABP4 and reduced glycolysis and mitochondrial respiration [39]. Astragalin showed inhibitory effect on oxidative stress induced aldose reductase activation in the kidneys of diabetic mice. On the other hand, astragalin may modulate mitochondria quality by activating the AMPK-dependent PGC1α pathway [40]. D-mannitol and kynurenic acid showed a regulatory role for enhancing lipid metabolism correlated with increasing PPARγ expression and stimulated lipid metabolism and thermogenics, respectively [41,42]. Chrologenic acid upregulated the expression of differentiation-related transcriptional factor PPARγ, mRNA levels of adipogenic transcriptional factors (Cebpb and Srebp1), and the lipolysis-related gene Hsl [43].

To investigate adipocyte differentiation and function in the presence of chemicals, we performed in silico studies to access effects of major components (Figure S2, Supplementary Materials) from leaf extract by targeting PPARγ, FABP4, and adiponectin proteins. When these major components docked as ligands to PPARγ protein (PDB ID 4EMA), all of them docked at the same location of the native ligand (BRL), indicating that our docking protocol was reliable. All ligands also displayed potential docked scores ranging from −8.4 to −7.4 (ΔG, kcal/mol) compared to those of a co-crystallized ligand (BRL) (−7.5 kcal/mol) (Figure 7).

**Figure 8:**
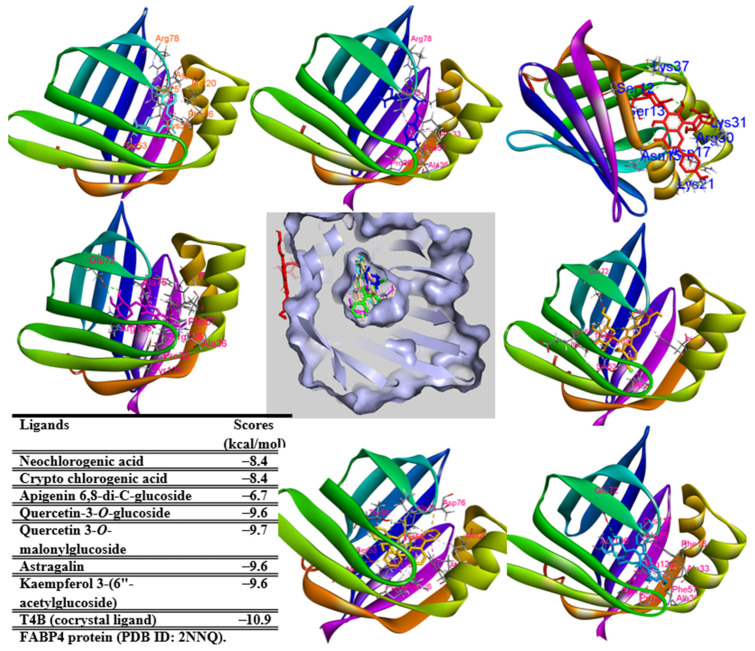
Molecular docking of compounds **10** (cyan), **18** (blue), **28** (red), **37** (light orange), **41** (light blue), **43** (yellow), **46** (magenta), and the co-crystallized ligand ((2′-(5-ethyl-3,4-diphenyl-1H-pyrazol-1-yl)-3-biphenylyl)oxy)acetic acid, docked into FABP4 protein (PDB ID 2NNQ).

Among interactions between ligand and protein in the binding pocket, amino acids such as TYR320, ASP396, ARG397, and GLN444 are key residues of the binding pocket of ligand–protein complexes (Figure S3, Supplementary Materials). Of the ligand complexes docked to FABP4 protein (PDB ID: 2NNQ), ligands **10**, **18**, **37**, **41**, **43**, **46**, and native ligand (T4B) docked into the same location in the binding pocket of ligand–protein complexes, whereas ligand **28** was located at another region of the complex. Therefore, compound **28** might not be well-correlated to 2NNQ protein. Indeed, the binding energy of ligands (**10**, **18**, **37**, **41**, **43**, and **46**) ranged from −9.7 to −8.7 (ΔG, kcal/mol), higher than that of the native ligand (−10.9 kcal/mol) but lower than that of ligand **28** (−6.7 kcal/mol) (Figure 8). Key amino acids such as PHE16, PHE57, LYS58, ARG78, ARG126, and TYR128 promoted interactions between ligand and protein in the binding pocket (Figure S4, Supplementary Materials). They were important residues of the complexes.

**Figure 9:**
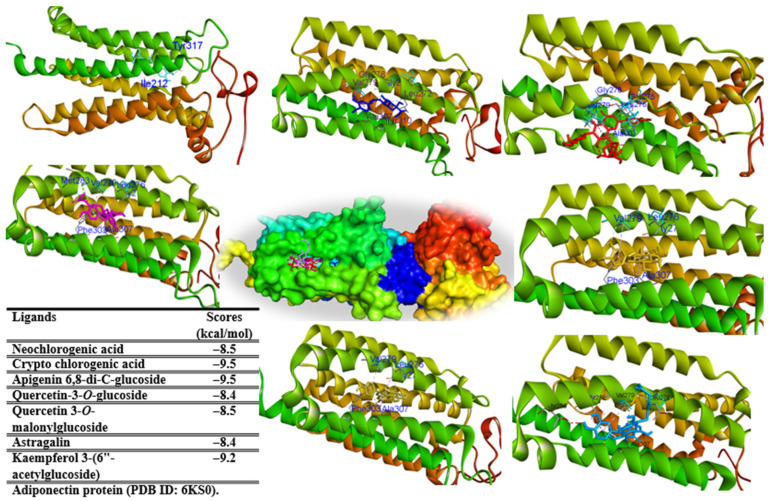
Molecular docking of compounds **10** (cyan), **18** (blue), **28** (red), **37** (light orange), **41** (light blue), **43** (yellow), **46** (magenta), docked into adiponectin protein (PDB ID: 6KS0).

When these ligands interacted with adiponectin (PDB ID: 6KS0) protein, all ligands displayed a low binding energy ranging from −9.5 to −8.4 kcal/mol (Figure 9). Among them, crypto chlorogenic acid (**18**) and apigenin 6,8-di-*C*-glucoside (**28**) exhibited the strong binding affinity through their interactions with key amino acids, including PHE271, GLY278, and TYR310 in the binding pockets of protein (Figure S5, Supplementary Materials).

## 3. Materials and Methods

### 3.1. Plant Materials

The different organs of *M*. *oleifera*, cultivated by Suncheonman Moringa union, were collected from Suncheon Bay in November 2022 and was identified by Professor Mina Lee (College of Pharmacy, Sunchon National University, Suncheon-si, Republic of Korea). The voucher specimens (SCNUP 37) were deposited at the laboratory of Pharmacognosy, College of Pharmacy, Sunchon National University, Suncheon-si, Jeonnam-do, Republic of Korea.

#### Extraction of Samples

The fresh materials of leaves, twigs, stem bark, seed, and roots of *M*. *oleifera* were dried in darkness at room temperature. Then, dried materials were ground into powder followed by sieving through a 250 μm^2^ sieve to ensure required sample homogeneity, before extracting with 75% EtOH by sonification for 90 min at room temperature. The extract solutions were dried under vacuum pressure. These total extracts of five organs of *M*. *oleifera* were stored in the refrigerator for future use.

### 3.2. Cell Culture

Mouse-derived 3T3-L1 preadipocytes are widely used as a model in obesity research because they differentiate consistently into mature adipocytes, making them suitable for studying adipogenesis, insulin response, lipid metabolism, and therapeutic development [17]. 3T3-L1 cells were purchased from the American Type Culture Collection (Manassas, VA, USA) and maintained in Dulbecco’s Modified Eagle’s Medium (DMEM; Welgene Inc., Deagu, Republic of Korea) supplemented with 10% bovine calf serum (Welgene), 100 U/mL penicillin, 100 μg/mL streptomycin, and 0.25 μg/mL amphotericin B at 37 °C under a 10% CO_2_. The HEK293a cell line (KCLB, Seoul, Republic of Korea) was maintained using DMEM containing 10% fetal bovine serum (FBS; Welgene, Republic of Korea), 100 U/mL penicillin, 100 μg/mL streptomycin, and 0.25 μg/mL amphotericin B at 37 °C under a 10% CO_2_. The medium was changed every 2 days and splitting was performed when the cells reached 70–80% confluency [44].

### 3.3. Adipocyte Differentiation

To induce differentiation, 3T3-L1 preadipocytes were seeded in 12−well culture plates at 5.0 × 10^4^ cells per well, cultured for two days to reach confluence and growth arrest (defined as day 0, D0), and then cultured for 48 h (D0 to D2) in differentiation medium containing DMEM supplemented with 10% FBS (Welgene), 0.5 mM IBMX (Sigma Aldrich, St Louis, MO, USA), 1 μM dexamethasone (Sigma Aldrich), and 1 μg/mL insulin (Sigma Aldrich). Cells were then cultured in a maintenance medium containing DMEM supplemented with 10% FBS and 1 μg/mL insulin. This medium was renewed every 48 h until day 6 (D6) as described previously [44,45].

### 3.4. Nile Red and Hoechst 33342 Staining

Differentiated 3T3-L1 cells were carefully washed twice with Dulbecco’s phosphate-buffered saline (DPBS) and fixed in 3.7% formalin for 30 min. The fixed cells were washed twice in DPBS and stained with 0.5 μg/mL Nile Red (Cayman Chemical, Ann Arbor, MI, USA) and 1 μg/mL Hoechst 33342 (Invitrogen, Carlsbad, CA, USA) for 10 min in the dark. The staining of lipid droplets and cell morphology were examined under a fluorescence microscope (NIB410, Nexcope, Ningbo, China) at ×200 magnification. All images were analyzed using ImageJ (NIH, Bethesda, MD, USA).

### 3.5. Quantitative Real-Time PCR Analysis

Total RNA was extracted using RiboExTM reagent (GeneAll Biotechnology, Seoul, Republic of Korea) and reverse transcribed into cDNA using the ReverTra AceTM qPCR RT kit (Toyobo, Osaka, Japan). Quantitative real-time PCR (qRT-PCR) was performed using a CFX Duet Real-Time PCR system (Bio-Rad, Hercules, CA, USA) and BioFACT™ 2X Real-Time PCR Master Mix (Biofact, Deajeon, Republic of Korea). The expression of 36B4 was measured as the internal control to calculate the relative expression levels of target genes. Most of the primer sequences used in the current experiments were used in our previous study [34]. The sequences of the remaining primers are as follows: PEPCK, (F) 5′-TCT CTG ATC CAG ACC TTC CAA-3′ and (R) 5′-GAA GTC CAG ACC GTT ATG CAG-3′; Plin2, (F) 5′-CCC TCA GCT CTC CTG TTA GG-3′ and (R) 5′-CAG AGG TCA CGG TCT TCA CG-3′.

### 3.6. Western Blotting Analysis

Cells were lysed in Mammalian Protein Extraction Reagent (Thermo Fisher Scientific, Rockford, IL, USA) containing Xpert protease inhibitor cocktail (GenDEPOT, Baker, TX, USA), and centrifuged at 12,000 rpm for 10 min at 4 °C. Supernatant proteins were separated on precast polyacrylamide gels (Bio-Rad) and subsequently electro-transferred onto polyvinylidene difluoride (PVDF) membranes. The membranes were blocked for 2 h in Tris-buffered saline with Tween 20 (TBST; containing 20 mM Tris-HCl, 150 mM NaCl, 0.2% Tween 20, pH 7.4) supplemented with 5% bovine serum albumin, and then incubated with an anti-PPARγ antibody (Cell Signaling Technology, Danvers, MA, USA, Cat. No. 2443), anti-adiponectin antibody (Thermo Fisher Scientific, Cat. No. MA1-054), and an anti-HSP 90 antibody (Santa Cruz Biotechnology, Dallas, TX, USA, Cat. No. SC-13119) at 4 °C overnight. After washing with fresh TBST, membranes were incubated with rabbit or mouse IgG conjugated to horseradish peroxidase (Bio-Rad, Cat. No. BR1706515 and BR1706516; 1:5000 dilution). Protein bands were visualized using ECL reagent (Bio-Rad) and an iBright CL1500 imaging system (Thermo Fisher Scientific) [46].

### 3.7. Luciferase and β-Galactosidase Assay

Luciferase assay was performed with HEK293a cell (KSLB, Seoul, Republic of Korea). Cells were seeded in 12−well culture plates at 1.8 × 10^5^ cells per well and the media was changed to plain media, GibcoTM Opti-Mem (Thermo Fisher Scientific), when confluency reached 80% at 20–22 h after seeding. Transfection was performed with Lipofectamine 3000 (Thermo Fisher Scientific) according to the manufacturer’s protocols. Cells were transfected with the mammalian expression vectors for pGL3−DR−1−basic, pCMV−β−galactosidase for transfection efficiency, and in the absence or presence of both PPARγ and RXRα. After 24 h, both Rosiglitazone (10 μM) or indicated concentrations (10, 30, and 100 μg/mL) of Moringa extracts (leaf, root, fruit, stem bark, twig) were treated for 24 h. Consequently, cell extracts were prepared with lysis buffer (25 mM Tris-phosphate (pH 7.8), 2 mM DTT, 1 mM CDTA, 10% Glycerol, and 1% Triton X−100), and the activities of luciferase and β-galactosidase were determined. These luminescence integrations were performed with MicroLumat Plus LB96V (Berthold Technologies, Bad Wildbad, Germany) and β-galactosidase activity was measured with Infinite^®^ 200 PRO (Tecan, Männedorf, Switzerland). The luciferase activity in relative light units was normalized to the β−galactosidase activity of each sample.

### 3.8. Analysis of Chemical Composition from Total Extracts of Leaves, Twigs, Stem Bark, Seeds, and Roots

The samples were analyzed using a Thermo Scientific Vanquish UHPLC system (Thermo Fisher Scientific, Sunnyvale, CA, USA) equipped with an autosampler, column temperature controller, and PDA detector. The mobile phases contained aqueous (water, A) and organic (ACN, B) buffered with 0.1% formic acid for both elements. The flow rate was set to 0.3 mL/min, and the injection volume was 2 μL. The gradient elution of the mobile phase was conducted as follows: 0 min (B: 4%), 1–4 min (B: 4–7%), 4–8 min (B: 7–10%), 8–15 min (B: 10–18%), 15–20 min (B: 18–50%), 20–23 min (B: 50–100%), 23–26 min (B: 100–100%), 26–27 min (B: 100–4%), and then maintained until the end of the process. The liquid chromatography was carried out on the same system with a Shimadu column and coupled with a Triple TOF 5600^+^ mass spectrometer (SCIEX, Foster, CA, USA) with a dousprayTM ion source. The mass parameter was set as follows: mass range (*m*/*z*) was from 50 to 1800, spray voltage was 5.5 kV (positive mode) and 4.5 kV (negative mode), temperature was 550 °C, ion source gas was 50 psi, curtain gas was 35 psi, declustering potential was 30 V, and collision energy was 10 V. The MS/MS parameter included collision energy at 35 V, and the collision energy spread was 15 V. Full scans and the data-dependent mode were selected to obtain the mass spectra data.

All compounds in the extracts of *M*. *oleifera* were tentatively identified by comparing the retention time, parent ion, and mass fragments in references or available standards.

### 3.9. Molecular Docking

The 3D structures of the PPARγ protein (PDB ID: 4EMA), FABP4 protein (PDB ID: 2NNQ), and adiponectin protein (PDB ID: 6KS0) were obtained from the RCSB protein data bank (https://www.rcsb.org; accessed on 8 August 2024). T4B and BRL co-crystal ligands were obtained from original proteins by using Discovery studio. Proteins and ligand were prepared using MGL tools 1.5.6. The structures of receptors were processed by removing water and adding polar hydrogen atoms and Kollman charges [47]. The structures of compounds (**10**, **28**, **37**, **41**, and **43**) were downloaded from PubChem (https://pubchem.ncbi.nlm.nih.gov, accessed on 10 August 2024) in sdf formats, and structures (**18** and **46**) were prepared using Avogadro package via the force field MMFF94 method. Then, the geometries of these structures of compounds were transformed into pdbqt format using Open Babel. Co-crystal ligand and the above compounds were obtained by adding Gasteiger charges, followed by torsion adjustment prior to applying the autogrid function. The grid box of coordinates was determined using Pymol to define the centroid of the native ligand in the crystal structures or key amino acids of the binding pocket of protein. The protein–ligand docking calculations were performed using AutoDock Vina 1.1.2. Each trial of Vina was run using an exhaustiveness of 8, while all other parameters were left as their default values. Residue–ligand interactions were visualized with Discovery studio 2021.

### 3.10. Statistical Analysis

All results are presented as mean ± standard error of the mean (SEM), while n denotes the number of culture wells analyzed. Two treatment groups were compared by an unpaired two-tailed Student’s *t*-test using Microsoft Excel version 2407-64 bit, while more than two groups were compared by analysis of variance with post hoc multiple comparison tests using GraphPad Prism. A *p* < 0.05 was considered statistically significant for all tests.

## 4. Conclusions

This study demonstrated that extracts from leaves, stem bark, and twigs of *M*. *oleifera* can significantly promote adipocyte differentiation in 3T3-L1 preadipocytes. These extracts increased intracellular lipid accumulation and upregulated genes of PPARγ, C/EBPα, adiponectin, and FABP4. The obtained results suggest that *M*. *oleifera* might modulate adipocyte differentiation and support the development of healthy adipose tissue, indicating its potential as a treatment for metabolic diseases. Given the critical role of adipocytes in glucose metabolism, *M. oleifera* extracts show promising applications in diabetes management by potentially enhancing insulin sensitivity, regulating lipid metabolism, and improving overall metabolic function. Additionally, the LC-MS/MS analysis of *M*. *oleifera* extracts revealed a diverse range of bioactive compounds, such as glycosides, flavones, fatty acids, and phenolics, many of which possess anti-inflammatory and antioxidant properties. Chronic inflammation and oxidative stress are key contributors to insulin resistance and metabolic syndrome. The ability of *M. oleifera* extracts to counteract these processes suggests additional benefits beyond regulating adipogenesis. By reducing inflammation and oxidative damage, *M. oleifera* extracts could help protect pancreatic β-cells, improve glucose uptake, and prevent the progression of insulin resistance into more severe forms of metabolic disease, such as type 2 diabetes. In silico studies supported these findings, showing that the major components of *M. oleifera* extracts bind to key proteins involved in metabolic regulation, such as PPARγ, FABP4, and adiponectin. These interactions provide insight into the molecular mechanisms by which *M. oleifera* extracts exerts their bioactivity, offering evidence for its use as a multi-target therapeutic agent. This is especially relevant for diabetes treatment which targets multiple pathways, such as glucose and lipid metabolisms and inflammation. The potential of *M. oleifera* extracts as an anti-diabetic agent is further supported by their ability to regulate critical signaling pathways involved in insulin sensitivity and lipid homeostasis. The upregulation of PPARγ and adiponectin, key regulators of glucose and lipid metabolism, suggests that *M. oleifera* extracts may improve insulin sensitivity and help restore normal glucose levels in patients with type 2 diabetes. Additionally, its action on SREBP1c, FAS, and other lipogenic genes indicates its ability to promote healthy adipose tissue formation, essential for maintaining metabolic balance. Furthermore, using *M. oleifera* extracts as a natural therapy offers the advantage of a potentially lower side effect profile compared to synthetic anti-diabetic drugs, such as thiazolidinediones, which are associated with weight gain and fluid retention. By leveraging the power of natural phytochemicals, *M. oleifera* extracts could serve as a safer alternative or complement to existing diabetes treatments. However, while the current findings are promising, further research is essential to isolate and characterize the specific bioactive compounds responsible for the anti-diabetic effects of *M. oleifera* extracts. In vivo studies are particularly needed to validate the efficacy and safety of these compounds, as well as to explore their long-term impact on glucose homeostasis and insulin sensitivity. Moreover, future studies should investigate the potential synergistic effects of *M. oleifera* extracts in combination with existing anti-diabetic therapies, which could lead to the development of more effective treatment regimens.

## Figures and Tables

**Table 1:** Identification of constituents from leaf, twig, stembark, root, and seed of *M*. *oleifera*.

No.	Compound	Leaves	Twigs	Stem Bark	Roots	Seeds	Formula	Adduct	*m*/*z* (Da)	RT (min)	Error (ppm)	Class
1	Glucose ^#^	−	+	−	+	+	C_6_H_12_O_6_	[M-H]^-^	179.05636	0.84	1.4	Glycoside
2	Mannitol ^#^	−	−	+	−	−	C_7_H_12_O_6_	[M-H]^-^	181.07231	0.88	0.2	Glycoside
3	Quinic acid ^#^	+	+	+	+	−	C_7_H_12_O_6_	[M-H]^-^	191.05626	0.93	0.8	Quinic
4	Unknown *	−	−	−	−	+	C_19_H_25_NO_19_	[M-H]^-^	341.10893	0.95	1.0	Unknown
5	Sucrose ^#^	+	+	−	+	+	C_12_H_22_O_11_	[M-H]^-^	341.10893	0.98	0.0	Glycoside
6	Citric acid ^#^	−	−	−	+	−	C_6_H_8_O_7_	[M-H]^-^	191.02004	1.21	1.6	Carboxylic acid
7	N-Acetyl-DL-glutamic acid ^#^	−	−	+	−	−	C_7_H_11_NO_5_	[M-H]^-^	188.05645	1.30	0.0	Amino acid
8	Adenosin ^#^	−	−	−	+	−	C_10_H_13_N_5_O_4_	[M+H]^+^	268.10422	1.32	0.7	Nucleoside
9	N-(1-Deoxy-1-fructosyl)phenylalanine ^#^	−	−	−	+	−	C_10_H_19_NO_7_	[M+H]^+^	328.13957	2.50	0.4	Phenolic glycoside
10	Neochlorogenic acid ^†^	+	+	−	−	−	C_16_H_18_O_9_	[M-H]^-^	353.08751	4.83	0.8	Quinic
11	Unknown *	−	−	+	−	−	C_14_H_20_O_8_	[M-H]^-^	315.10825	4.89	0.9	Unknown
12	Kynurenic acid ^#^	−	−	+	−	−	C_10_H_7_NO_3_	[M-H]^-^	188.03538	5.29	0.4	Alkaloid
13	2-Isopropylmalic acid ^#^	−	−	−	+	−	C_7_H_12_O_5_	[M-H]^-^	175.0615	5.34	1.7	Carboxylic acid
14	Unknown *	−	−	+	−	−	C_13_H_16_O_7_	[M-H]^-^	283.0822	5.65	0.4	Unknown
*15*	*p*-Coumaroylquinic acid ^#^	+	+	−	−	−	C_16_H_18_O_8_	[M-H]^-^	337.09319	6.21	0.9	Quinic
16	Chlorogenic acid ^†^	+	−	−	−	−	C_16_H_18_O_9_	[M-H]^-^	353.08745	6.90	1.0	Quinic
17	3-Methoxytyrosine ^#^	−	+	−	+	−	C_10_H_13_NO_4_	[M-H]^-^	210.07746	7.49	1.3	Phenolic
18	Cryptochlorogenic acid ^†^	+	+	−	−	−	C_16_H_18_O_9_	[M-H]^-^	353.08751	7.53	0.8	Quinic
19	Unknown *	−	−	−	+	−	C_19_H_27_NO_11_	[M-H]^-^	444.15071	7.55	1.0	Unknown
20	Unknown *	−	−	−	+	−	C_18_H_29_NO_10_	[M+H]^+^	420.18629	8.21	0.3	Unknown
21	Acetyl-4-(*α*-L-rhamnopyranosyloxy) benzyl glucosinolate ^ϕ^	+	−	−	−	−	C_22_H_31_NO_15_S_2_	[M-H]^-^	612.10619	8.26	0.1	Phenolic glycoside
22	Unknown *	−	−	+	−	−	C_14_H_18_O_8_	[M-H]^-^	313.09267	8.56	0.7	Unknown
23	Orcinol gentiobioside *	+	+	+	+	+	C_19_H_28_O_12_	[M-H]^-^	447.15036	8.81	1.0	Phenolic glycoside
24	4-Methylumbelliferyl-*α*-D-galactopyranoside *	−	+	−	−	−	C_16_H_18_O_8_	[M-H]^-^	337.09254	8.97	1.0	Phenolic glycoside
25	Pantothenic acid ^#^	−	+	+	+	−	C_9_H_17_NO_5_	[M-H]^-^	218.10356	9.10	0.8	Alkaloid
26	4-Hydroxyhydrocinnamic acid *	−	−	+	−	−	C_9_H_10_O_3_	[M+H]^+^	165.05603	9.68	1.9	Phenolic
27	2-Formylphenyl 2-acetamido-2-deoxy-*β*-D-glucopyranoside *	+	+	+	−	+	C_15_H_19_NO_7_	[M-H]^-^	324.10871	10.34	0.5	Phenolic glucoside
28	Apigenin-6,8-di-*C*-glucoside (Vitexin-2) ^#^	+	+	+	−	−	C_27_H_30_O_15_	[M-H]^-^	593.15097	10.79	0.4	Flavone-di-*C*-glycosides
29	Acetyl-L-phenylalanine ^#^	−	−	+	−	−	C_11_H_13_NO_3_	[M-H]^-^	206.08244	10.91	0.8	Amino acid
30	Suberic acid ^#^	−	−	+	−	−	C_8_H_14_O_4_	[M-H]^-^	173.08207	11.13	0.8	Fatty acid
31	Nonioside D *	+	+	−	−	−	C_18_H_32_O_12_	[M-H]^-^	439.18181	12.2	0.7	Butanolic acid glycosides
32	Unknown *	−	+	−	−	−	C_20_H_25_NO_9_	[M-H]^-^	422.14524	12.65	1.0	Unknown
33	9-Oxo-nonanoic acid	−	−	+	−	−	C_9_H_16_O_3_	[M-H]^-^	171.1024	13.59	0.8	Fatty acid
34	Vitexin ^#^	+	+	−	−	−	C_21_H_20_O_10_	[M-H]^-^	431.09828	14.46	0.2	Flavone-*C*-glucose
35	Rutin ^#^	+	+	−	−	−	C_27_H_30_O_16_	[M-H]^-^	609.14672	14.92	1.0	Flavone-Di-*O*-glycosides
36	Isovitexin ^#^	+	+	−	−	−	C_21_H_20_O_10_	[M-H]^-^	431.09859	14.95	0.5	Flavone-Di-*O*-glycosides
37	Quercetin-3-glucoside ^†^	+	−	−	+	−	C_21_H_20_O_12_	[M-H]^-^	463.0886	15.36	0.9	Flavone glucoside
38	9-[2,3-Dihydroxypropoxy]-9-oxononanoic acid ^#^	−	−	+	+	−	C_12_H_22_O_6_	[M-H]^-^	261.13411	15.72	1.0	Fatty acid
39	Quercetin 3-(6″-acetylglucoside) ^#^	−	+	−	−	−	C_23_H_22_O_13_	[M-H]^-^	505.09856	15.98	0.4	Flavone glucoside
40	Azelaic acid ^#^	−	+	+	+	+	C_9_H_16_O_4_	[M-H]^-^	187.09775	16.34	0.9	Fatty acid
41	Quercetin 3-*O*-malonylglucoside ^#^	+	+	−	−	−	C_24_H_22_O_15_	[M-H]^-^	549.08917	16.74	1.0	Flavone-*O*-glucoside
42	Kaempferol-3-rutinoside ^#^	+	+	−	−	−	C_27_H_30_O_15_	[M-H]^-^	593.1516	17.09	0.7	Flavone-*O*-glycoside
43	Astragalin ^†^	+	+	−	+	−	C_27_H_30_O_15_	[M-H]^-^	447.09308	17.58	0.5	Flavone-*O*-glycoside
44	Isorhamnetin-3-glucoside ^#^	+	+	−	+	−	C_22_H_22_O_12_	[M-H]^-^	477.10425	18.25	0.8	Flavone-*O*-glycoside
45	Secoisolariciresinol ^#^	−	−	−	+	−	C_20_H_26_O_6_	[M-H]^-^	361.16596	18.63	0.8	Phenolic
46	Kaempferol 3-(6″-acetylglucoside) ^ϕ^	+	+	−	−	−	C_22_H_22_O_12_	[M-H]^-^	489.10414	19.38	0.6	Flavone-*O*-glycoside
47	Unknown *	−	+	−	−	−	C_12_H_17_NO_4_	[M-H]^-^	238.10863	19.77	0.6	Unknown
48	Decanedioic acid ^#^	−	−	+	−	−	C_10_H_18_O_4_	[M-H]^-^	201.11326	21.56	0.1	Fatty acid
49	9,12,13-Trihydroxy-10,15-octadecadienoic acid ^#^	−	−	−	+	−	C_18_H_32_O_5_	[M-H]^-^	327.21799	22.47	0.9	Fatty acid
50	Tridecanedioic acid	+	−	−	−	−	C_13_H_24_O_4_	[M-H]^-^	243.1588	22.937	0.3	Fatty acid

^#^ In-house MS/MS library and online database such as GNPS, MASS bank, or Metlin. ^†^ Reference standard. ^ϕ^ [25]. * Extract MS with isotope mass. “+” and “−”: detected and not detected in chromatograms, respectively.

## Data Availability

Data are contained within the article and the Supplementary Materials.

## Supplementary Materials

The following supporting information can be downloaded at: https://www.mdpi.com/article/10.3390/ph17101310/s1. Figure S1: Inhibitory effects of each Moringa extracts on adipocyte differentiation; Figure S2: The structures of major peaks were detected from leaf extract of *M*. *oleifera*; Figure S3: Interactions of compounds **10** (cyan), **18** (blue), **28** (red), **37** (light orange), **41** (light blue), **43** (yellow), and **46** (magenta) with amino acid when they were docked into PPARγ protein (PDB ID: 4EMA); Figure S4: interactions of compounds **10** (cyan), **18** (blue), **28** (red), **37** (light orange), **41** (light blue), **43** (yellow), and **46** (magenta) with amino acid when they were docked into FABP4 protein (PDB ID 2NNQ); Figure S5: Interactions of compounds **10** (cyan), **18** (blue), **28** (red), **37** (light orange), **41** (light blue), **43** (yellow), and **46** (magenta) with amino acid when they were docked into adiponectin protein (PDB ID: 6KS0); Table S1: In silico docking scores and interactions of ligands and proteins.
