# Supplementary material for: Chemical Investigation and Regulation of Adipogenic Differentiation of Cultivated Moringa oleifera
Source: Pharmaceuticals (Basel). 2024 Oct 1;17(10):1310. doi: 10.3390/ph17101310 (PMC11510418; doi:10.3390/ph17101310)
Supplement: Supplementary file 1 [file pharmaceuticals-17-01310-s001.zip › pharmaceuticals-3192837-supplementary.pdf]

Supplementary materials

Table of Contents

Figure S1. Inhibitory effects of each *Moringa* extracts on adipocyte differentiation 1

Figure S2. The structures of major peaks were detected from leaf extract of *M. oleifera* 2

Table S1. *In-silico* docking scores and interactions of ligands and proteins 3

Figure S3. Interactions of compounds **10** (cyan), **18** (blue), **28** (red), **37** (light orange), **41** (light blue), **43** (wheat), **46** (magenta), with amino acid when they were docked into PPARγ protein (**PDB ID: 4EMA**). 4

Figure S4. Interactions of compounds **10** (cyan), **18** (blue), **28** (red), **37** (light orange), **41** (light blue), **43** (wheat), **46** (magenta), with amino acid when they were docked into FABP4 protein (**PDB ID 2NNQ**). 4

Figure S5. Interactions of compounds **10** (cyan), **18** (blue), **28** (red), **37** (light orange), **41** (light blue), **43** (wheat), **46** (magenta), with amino acid when they were docked into adiponectin protein (**PDB ID: 6KS0**). .5

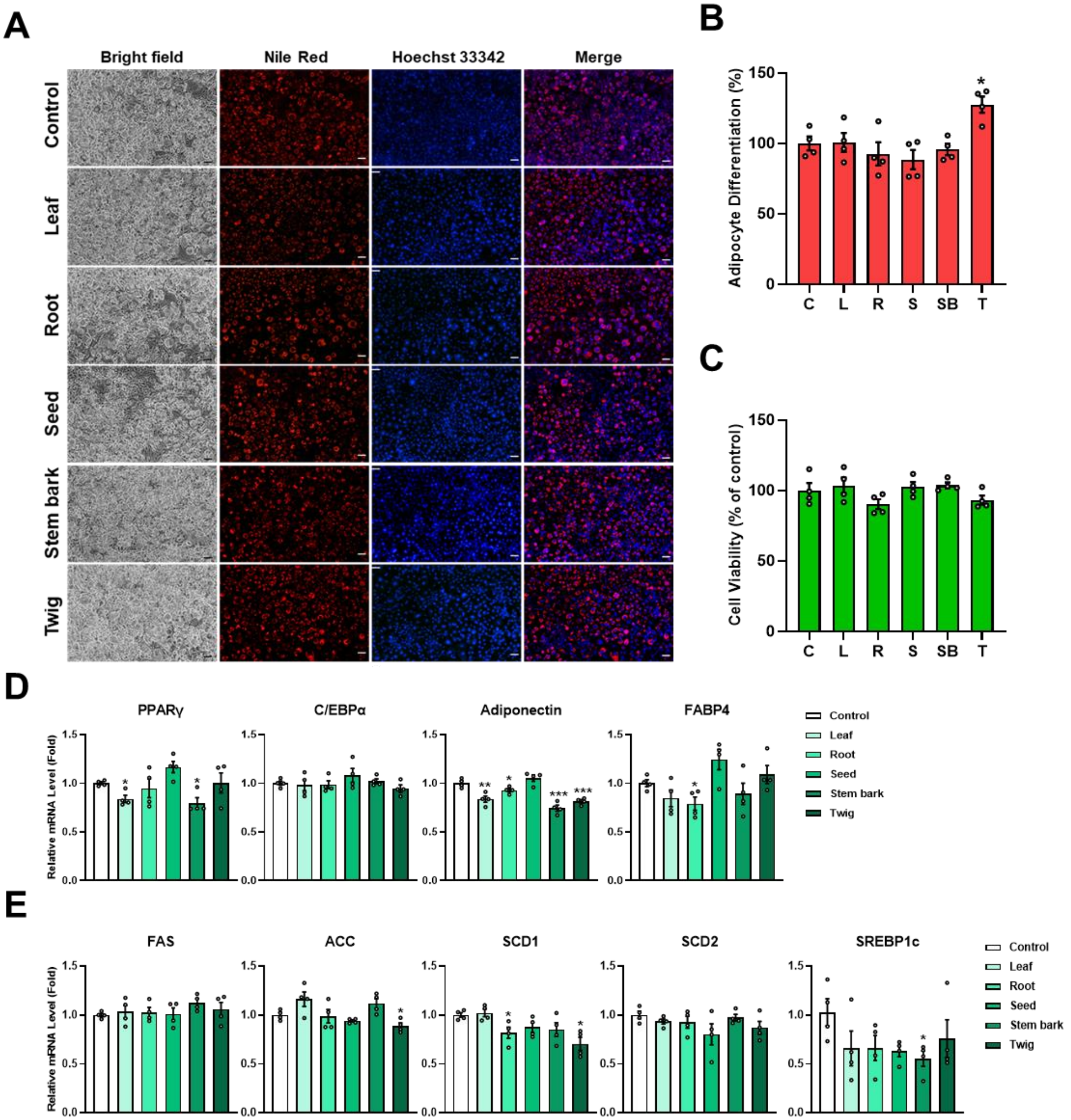

Figure S1. Inhibitory effects of each *Moringa* extracts on adipocyte differentiation. 3T3-L1 preadipocytes were cultured in a 1X differentiation induction medium for 6 days, either without (control) or with each Moringa extracts (100 μg/ml). (A) Cells were differentiated for 6 days were then stained with Nile Red and Hoechst 33342. Images were acquired by epifluorescence microscopy. Scale bar = 100 μm. (B and C) Quantification of Nile Red intensity and cell viability was performed using ImageJ software. (D) mRNA expression levels of adipogenic genes were measured by qRT-PCR analysis. Data are shown as mean ± SEM (n = 4 per group). (E) The protein expression levels of PPARγ and Adiponectin were analyzed using Western blotting. Results are shown as mean ± SEM (n = 2 per group). (F) mRNA expression levels of

lipogenic genes were measured by qRT-PCR. Relative mRNA expression levels were normalized to 36B4 and are shown as mean ± SEM (n = 4 per group). All data are indicated as *p < 0.05, **p < 0.01, and ***p < 0.001 compared to control.

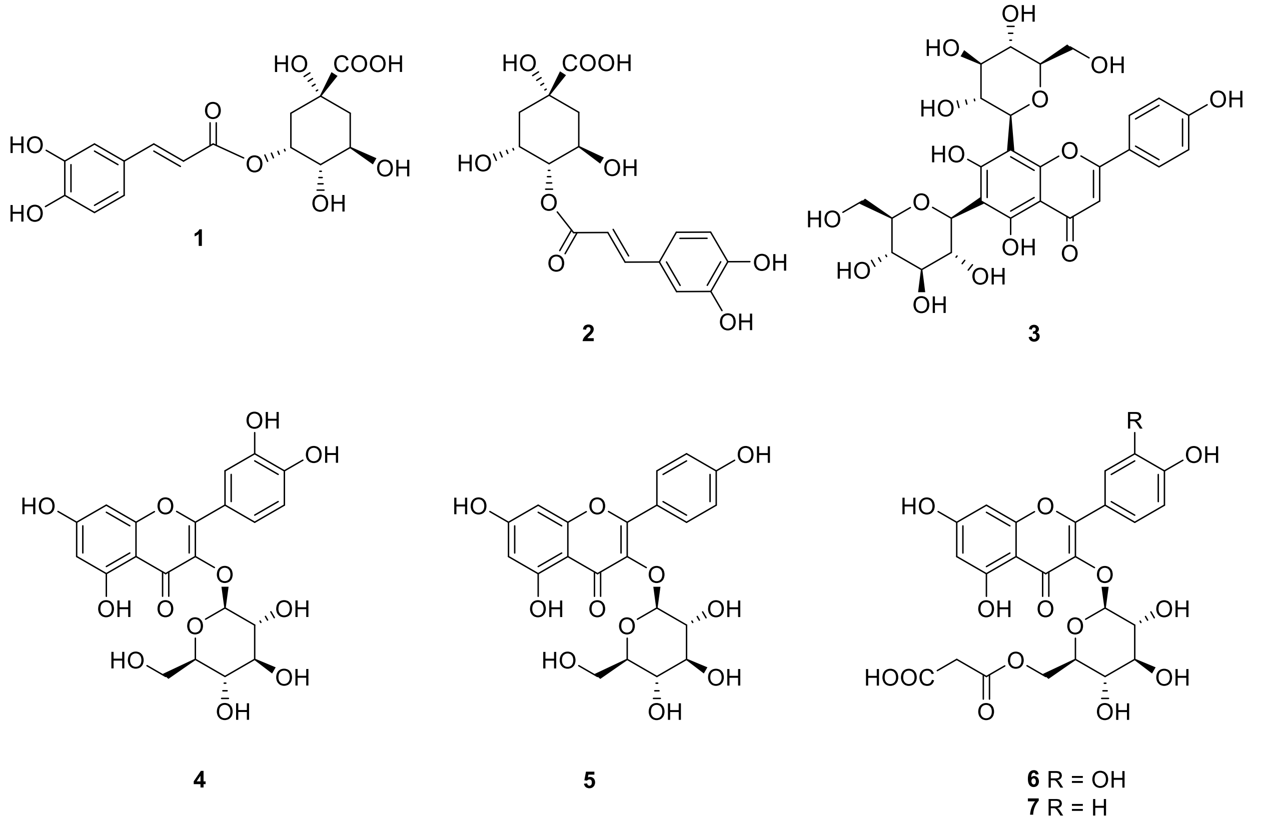

Figure S2. The structures of major peaks were detected from leaf extract of *M. oleifera*.

Table S1. *In-silico* docking scores and interactions of ligands and proteins.

	PPARγ (PDB ID 4EMA		FABP4 (PDB ID: 2NNQ)		Adiponectin (PDB ID: 6KS0)	
Ligand	Score (kcal/mol)	Key interaction	Score (kcal/mol)	Key interaction	Score (kcal/mol)	Key interaction
**10**	-7.4	TYR320, ASP441, ARG443, GLN444	**-8.4**	LYS58, ASP76 ARG78	**-8.5**	TYR317,
**18**	-7.9	TYR320, ARG443, GLN444	**-8.4**	TYR19, ARG78	**-9.5**	GLY278, VAL279, TYR310, ALA307
**28**	-8.0	GLU369, ARG397, GLN444, GLU448	**-6.7**	ASN15, LYS37	**-9.5**	GLY275, GLY278, VAL279, ALA307,
**37**	-8.1	ASP396, ARG397, GLN444	**-9.6**	PHE16, ILE51, GLU72, ARG106	**-8.4**	GLY275, ALA307, VAL279
**41**	-8.4	TYR320, ASP396, ARG397, GLN444	**-9.7**	PHE16, ALA36, PHE57, GLU72, ARG106, ARG126, TYR128	**-8.5**	GLY275, LEU276, VAL279, ALA307,
**43**	-8.0	GLU324, LYS319, GLN444	**-9.6**	PHE16, MET20, SER55, LYS58, THR60, ARG78, VAL115	**-8.4**	GLY275, VAL279, ALA307,
**46**	-8.7	ASP396, ARG397, ASP441, GLN444, GLU448	**-9.6**	PHE16, TYR19, PHE57, ARG106, ARG126, TYR128	**-9.2**	GLY275, VAL279, MET283, ALA307
**BRL**	**-7.5**	TYR320, ASP441	-	-	-	-
**T4B**	-	-	**-10.9**	PHE16, TYR19, MET20, PHE57, LYS58, THR60, VAL115, ARG126, TYR128	-	-

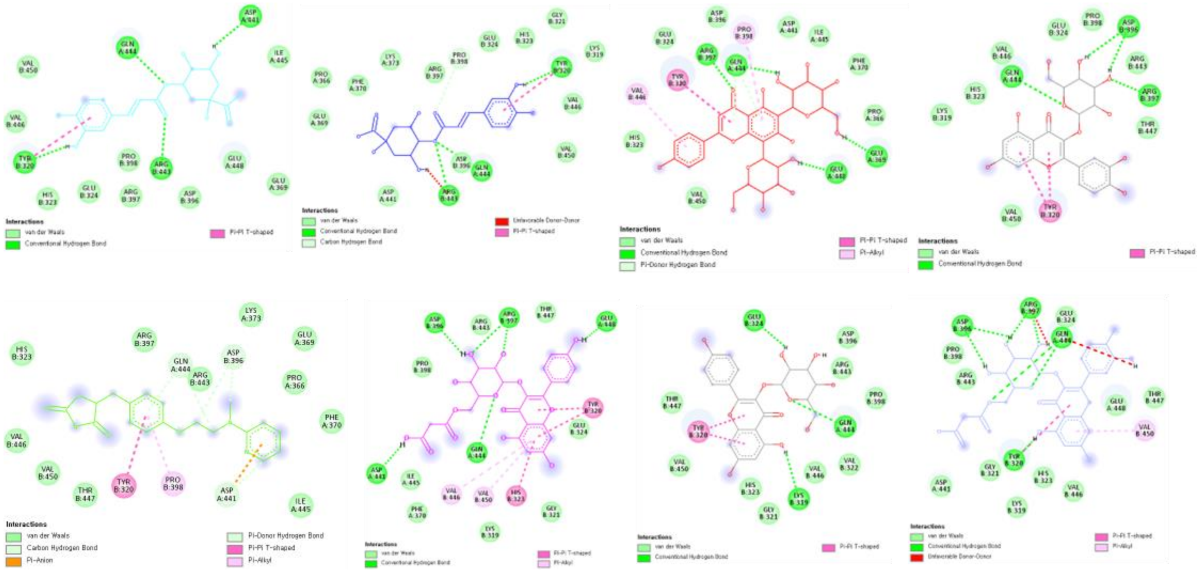

Figure S3. interactions of compounds **10** (cyan), **18** (blue), **28** (red), **37** (light orange), **41** (light blue), **43** (wheat), **46** (magenta), with amino acid when they were docked into PPARγ protein (**PDB ID: 4EMA**).

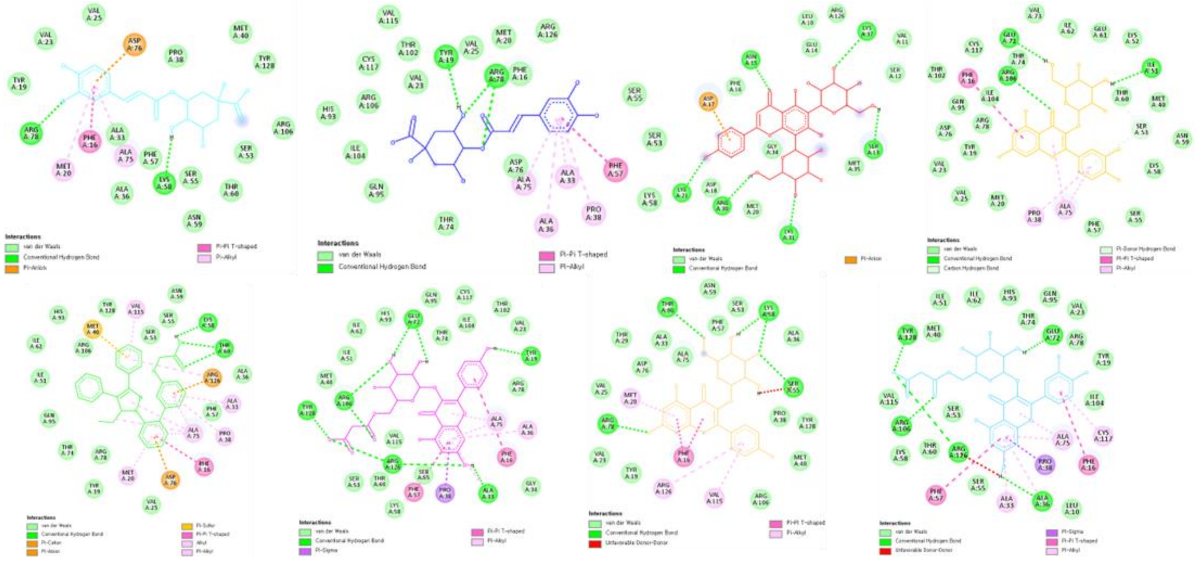

Figure S4. interactions of compounds **10** (cyan), **18** (blue), **28** (red), **37** (light orange), **41** (light blue), **43** (wheat), **46** (magenta), with amino acid when they were docked into FABP4 protein (**PDB ID 2NNQ**).

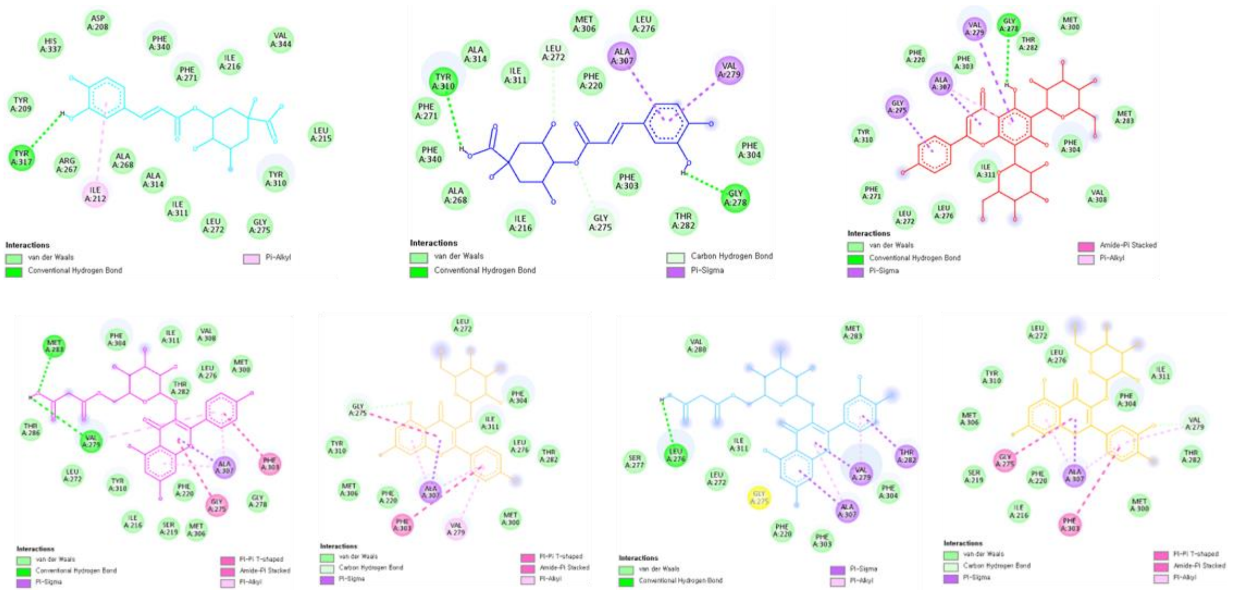

Figure S5. interactions of compounds **10** (cyan), **18** (blue), **28** (red), **37** (light orange), **41** (light blue), **43** (wheat), **46** (magenta), with amino acid when they were docked into adiponectin protein (**PDB ID: 6KS0**).
